# Broadband antireflective silicon nanostructures produced by spin-coated Ag nanoparticles

**DOI:** 10.1186/1556-276X-9-54

**Published:** 2014-02-01

**Authors:** Joon Beom Kim, Chan Il Yeo, Yong Hwan Lee, Sooraj Ravindran, Yong Tak Lee

**Affiliations:** 1 School of Information and Communications, Gwangju Institute of Science and Technology, 1 Oryong-dong, Buk-gu, Gwangju, 500-712, Republic of Korea; 2 Advanced Photonics Research Institute, Gwangju Institute of Science and Technology, 1 Oryong-dong, Buk-gu, Gwangju, 500-712, Republic of Korea

**Keywords:** Silicon nanostructures, Spin-coated Ag nanoparticles, Antireflection, Rigorous coupled-wave analysis

## Abstract

We report the fabrication of broadband antireflective silicon (Si) nanostructures fabricated using spin-coated silver (Ag) nanoparticles as an etch mask followed by inductively coupled plasma (ICP) etching process. This fabrication technique is a simple, fast, cost-effective, and high-throughput method, making it highly suitable for mass production. Prior to the fabrication of Si nanostructures, theoretical investigations were carried out using a rigorous coupled-wave analysis method in order to determine the effects of variations in the geometrical features of Si nanostructures to obtain antireflection over a broad wavelength range. The Ag ink ratio and ICP etching conditions, which can affect the distribution, distance between the adjacent nanostructures, and height of the resulting Si nanostructures, were carefully adjusted to determine the optimal experimental conditions for obtaining desirable Si nanostructures for practical applications. The Si nanostructures fabricated using the optimal experimental conditions showed a very low average reflectance of 8.3%, which is much lower than that of bulk Si (36.8%), as well as a very low reflectance for a wide range of incident angles and different polarizations over a broad wavelength range of 300 to 1,100 nm. These results indicate that the fabrication technique is highly beneficial to produce antireflective structures for Si-based device applications requiring low light reflection.

## Background

Silicon (Si) is an important material used for optoelectronic device applications, such as sensors, photodetectors, and solar cells, due to its abundance in the earth's crust, low-cost, and mature fabrication technique [1-4]. For these devices, minimizing the light reflection on the surface thereby increasing the light transmission into the device is the key to increase the device performance. However, more than 30% of the incident light is lost through Fresnel reflection owing to the large refractive index difference between air (*n*_air_ = 1) and Si (*n*_Si_ approximately 3.8), and therefore, antireflective structures are indispensible to improve the device performance. Conventional multilayered thin-film antireflection coatings have been widely used to suppress the unwanted surface reflection losses. However, these coatings have serious drawbacks that are related to material selection, mechanical instability, and thermal mismatch. Furthermore, these antireflective coatings can suppress the reflections only over a narrow wavelength and incident angle range [5,6]. Recently, bioinspired antireflective nanostructures with tapered features have attracted great interest for improving the performance of optical and optoelectronic devices due to their broadband and omnidirectional antireflection properties as well as long-term stability [1,5-13]. A commonly used technique to produce such antireflective nanostructures on various materials is dry etching of nano-scale etch masks formed by electron-beam or interference lithography process [5,6,9,10]. However, lithography-based nanopatterning method is not suitable for mass production because it is a time-consuming process requiring delicate and expensive equipment, reducing the cost effectiveness. Numerous research efforts have therefore been carried out to form nano-scale etch masks using a simple, fast, and cost-effective nanopatterning method in order to enhance productivity and thereby reduce the fabrication cost of antireflective nanostructures.

In this paper, we report a simplified fabrication technique for producing antireflective nanostructures having tapered profile on Si substrates without using any lithography steps. To achieve this goal, nano-scale silver (Ag) etch masks were formed using spin-coating Ag ink and subsequent sintering process. The significant advantage of the reported technique is that it requires only a low temperature and a short process duration to form the Ag etch masks [7,11,12]. Furthermore, the technique avoids the usage of any lithographic process, making it highly cost-effective for mass production [8]. Prior to fabrication, the period- (i.e., distance between the adjacent nanostructures) and height-dependent reflection characteristics of the Si nanostructures were theoretically investigated using a rigorous coupled-wave analysis (RCWA) method in order to provide a guideline for producing a desirable Si nanostructure with broadband antireflection properties because the antireflection properties of these nanostructures are closely correlated with their geometry [6-12]. The Ag ink ratio and dry etching conditions, which affect the distribution, distance between adjacent nanostructures, and height of resulting Si nanostructures, were carefully adjusted, and optimal experimental conditions were found that can produce desirable antireflective Si nanostructures for practical applications. We found that the fabricated Si antireflective nanostructures have excellent antireflective properties over a wide wavelength range and polarization-independent antireflection properties.

## Methods

### Optical modeling of Si nanostructures

**Figure 1:**
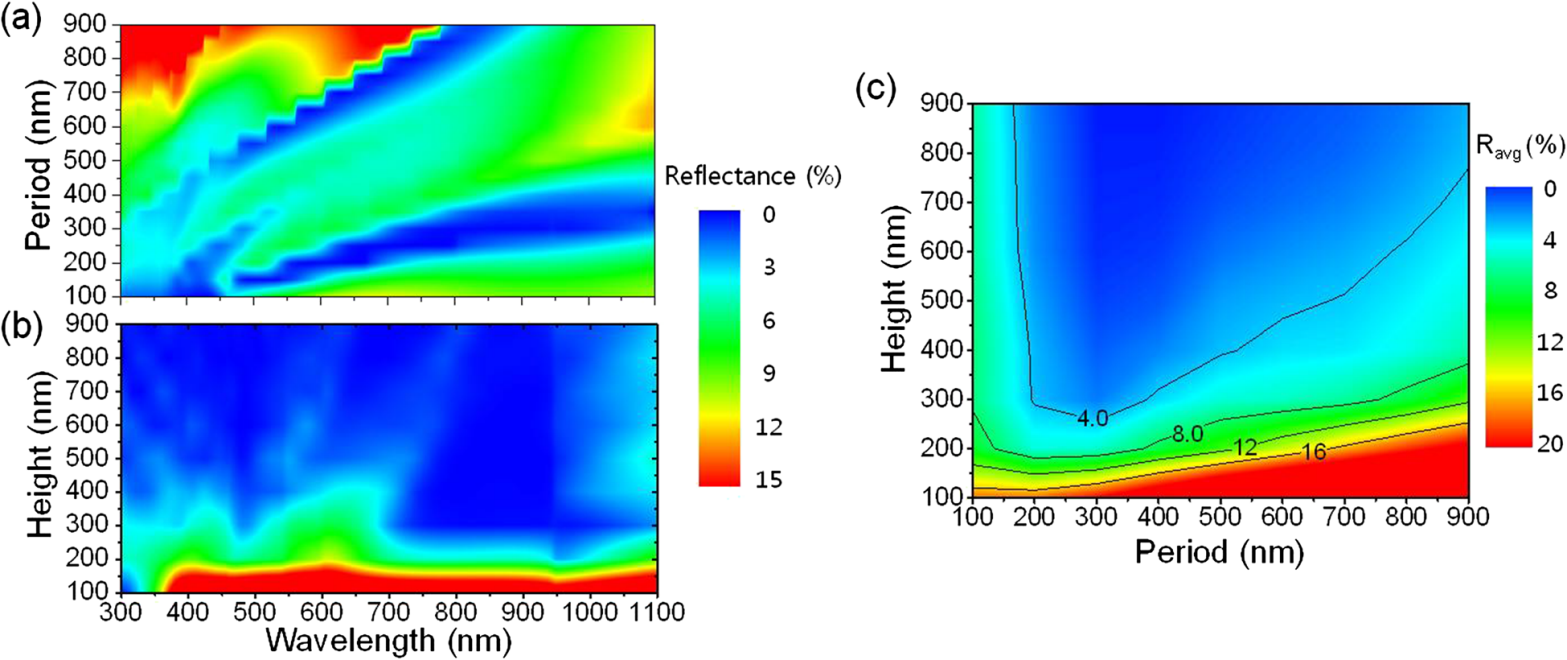
**Calculated reflectance of Si nanostructures.** Calculated **(a)** period- (i.e., distance between adjacent nanostructures) and **(b)** height-dependent reflectance of Si nanostructures as a function of wavelength when the height and period were fixed at 300 nm, respectively. **(c)** Calculated average reflectance as functions of period and height of the Si nanostructures in a wavelength range of 300 to 1,100 nm. The bottom diameter to period ratio and the top diameter to period ratio of the Si nanostructures used in the simulation were assumed as 0.8 and 0.15, respectively.

Closely packed nanostructures with short periods and larger heights considerably lower the reflection; however, the fabrication processes required to realize such nanostructures are complex and expensive [9,10]. Thus, based on theoretical calculations, it is necessary to determine the period and height of the nanostructure that can be fabricated at ease using the proposed technique to achieve desirable antireflection properties. For practical applications such as solar cells, it is important that the nanostructures have a low reflectance over a broad wavelength range. To determine the desirable geometric features (i.e., period and height) for Si nanostructures that can achieve broadband antireflection for practical applications, we conducted a theoretical investigation of the reflectance behavior using the RCWA method [14]. To calculate the reflectance, a truncated cone-shaped Si nanostructure with a bottom diameter to period ratio of 0.8 and a top diameter to period ratio of 0.15 was assumed in order to simplify the calculations. The simulation model was constructed based on previous experimental results which used metal nanoparticles as a dry etching mask [8,11,12]. Figure 1a shows the calculated reflectance of the Si nanostructures for various periods for a fixed height of 300 nm. The overall reflectance at first somewhat decreased with an increasing period and then began to increase as the period was further increased. We also observed that there were regions with low reflectance (<3%) over a broad wavelength range, when the period was around 200 to 400 nm. This indicates that the selection of proper period is essential to obtain nanostructures with broadband antireflection properties. Figure 1b shows the calculated height-dependent reflectance of the Si nanostructures when their period was fixed at 300 nm. It is clear that the reflectance decreased considerably with an increasing height. Although structures with taller height exhibits lower reflectance, a ‘too tall’ height is not favorable because it can cause mechanical instability [8,9]. Hence, choosing the proper height for antireflective nanostructures is necessary for practical applications. To precisely determine the proper period and height of antireflective Si nanostructures for practical applications, the average reflectance was calculated in the wavelength range of 300 to 1,100 nm for various periods and heights. Figure 1c shows the contour plot of the calculated average reflectance of the antireflective nanostructures as functions of the period and height. When the height of the Si nanostructures was approximately 400 nm, the Si nanostructures having a period between 200 to 500 nm (i.e., an aspect ratio of <2) exhibited a very low average reflectance of <4%. We thus selected the height of Si nanostructures to be <400 nm and the period in the range of 200 to 500 nm as the geometric parameters that are practically feasible for achieving broadband antireflection based on RCWA analysis.

### Fabrication of Si nanostructures

**Figure 2:**
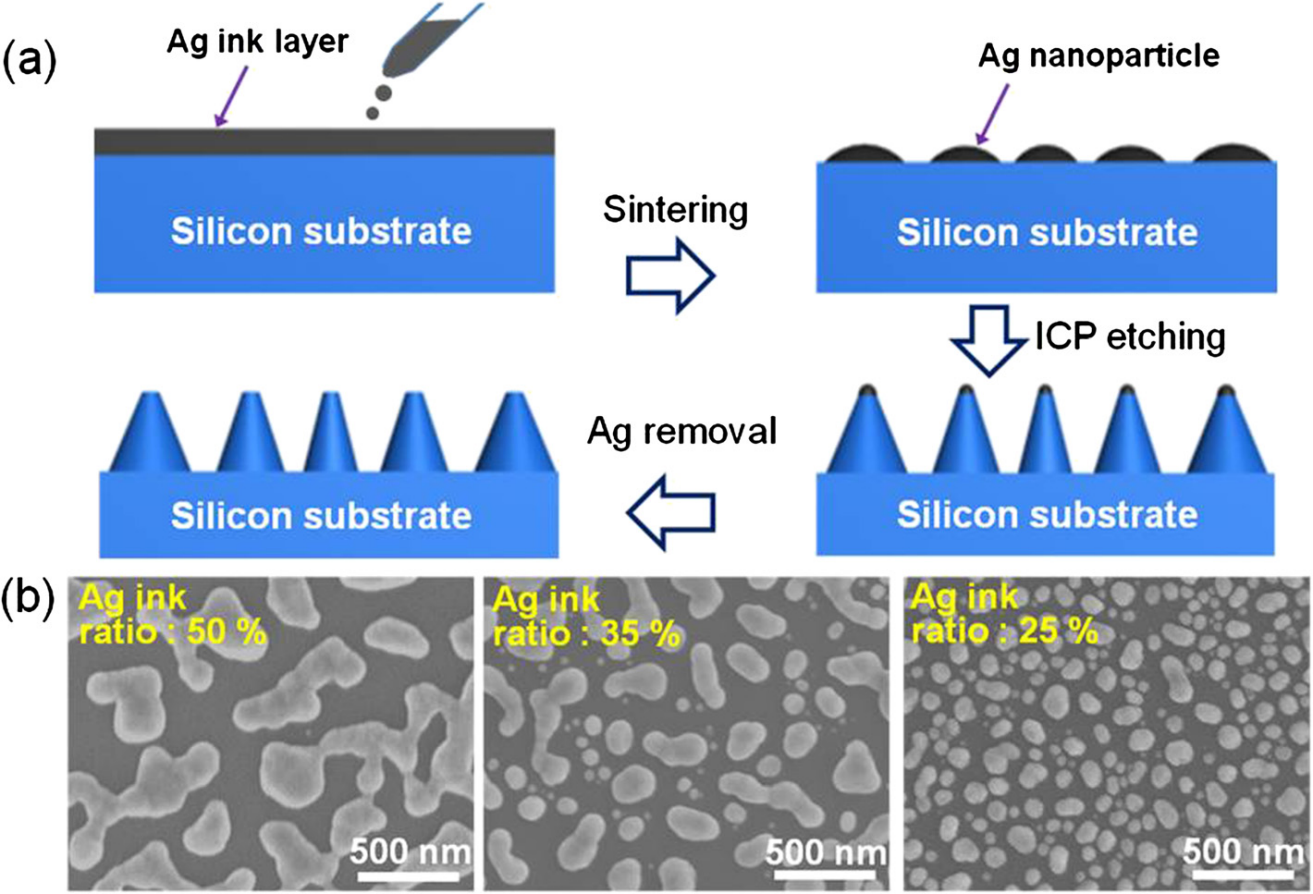
**Process steps to fabricate Si nanostructures and Ag ink ratio-dependent distribution of Ag nanoparticles. (a)** Fabrication procedure for forming Si nanostructures using spin-coated Ag ink nanoparticles and subsequent ICP etching. **(b)** Top-view SEM images of the randomly distributed Ag nanoparticles on Si substrate. The corresponding Ag ink ratios used are shown in the inset.

Figure 2a shows a schematic illustration of the process steps to fabricate antireflective nanostructures on a Si substrate by inductively coupled plasma (ICP) etching using spin-coated Ag nanoparticles as the etch mask. The spin-coating process was performed at 5,000 rpm for 20 s, and the sintering process was carried out at 250°C on a hotplate for 5 min in order to transform the as-coated Ag ink layer into nano-scale Ag etch masks. During the sintering process, the solvent-based Ag ink, which consisted of soluble Ag clusters containing Ag atoms of 10 wt.%, randomly agglomerated to reach an energetically stable state [7,8,15]. For this reason, the sintering temperature was carefully chosen. It is worth noting that the temperature and process time to make Ag nanoparticles is much lower and shorter, respectively, than the previously reported method in which metal nanoparticles were formed through thermal dewetting of evaporated thin metal film [7,11,12,15]. The Ag ink ratio in a mixture of Ag ink and isopropanol was adjusted to produce differently distributed Ag nanoparticles because their distribution predominantly determines the distribution of the resulting nanostructures, which strongly affects their antireflection properties [6-8,12]. Figure 2b shows the top-view field-emission scanning electron microscope (FE-SEM, S-4700, Hitachi, Ltd., Tokyo, Japan) images of the randomly distributed Ag nanoparticles formed on the Si substrate for various Ag ink ratios. As the Ag ink ratio was decreased, the size and the distance between adjacent Ag nanoparticles became smaller and closer, respectively, as can be seen in Figure 2b. The fractional surface coverage of Ag nanoparticles on Si substrate also decreased from 54.2% to 40.3% when Ag ink ratio was decreased from 50% to 25%. This can be attributed to the reduced quantity of Ag atoms in the spin-coated Ag ink due to dilution. We calculated the average distance between adjacent Ag nanoparticles, which in turn affect the distance between adjacent Si nanostructures, using a free-ware image processing program (ImageJ 1.42q, NIH). The calculated average distance between adjacent Ag nanoparticles formed using Ag ink ratio of 35% and 25% showed an average distance of 358 ± 32 and 184 ± 13 nm, respectively. To produce Si nanostructures using the Ag nanoparticles, dry etching was carried out using an ICP etcher (Plasmalab System 100, Oxford Instrument Co., Oxford, UK). ICP etching conditions, including the radio-frequency (RF) power, flow rate of Ar gas, and etching time, were carefully adjusted in an SiCl_4_ plasma to obtain the desire antireflective Si nanostructures. The ICP power, process pressure, and flow rate of SiCl_4_ were fixed at 0 W, 2 mTorr, and 5 sccm, respectively. After the ICP etching, the samples were soaked in a chemical etchant mixture containing KI, I_2_, and deionized (DI) water at room temperature for 5 s to remove the residual Ag nanoparticles. Finally, the samples were rinsed with DI water and dried with N_2_ jet.

## Results and discussion

**Figure 3:**
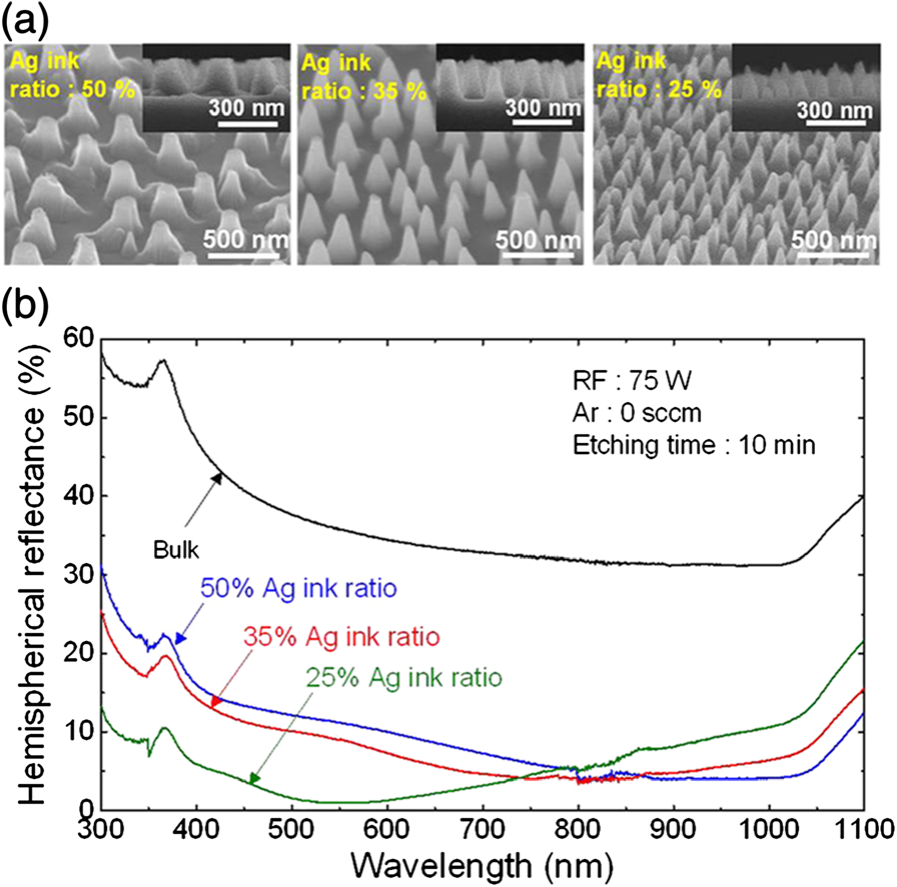
**SEM images of the Si nanostructures and the measured hemispherical reflectance spectra. (a)** Forty-five-degree-tilted-view SEM images and **(b)** hemispherical reflectance of the fabricated Si nanostructures corresponding to Ag ink ratios of 25%, 35%, and 50%.

Figure 3a shows the 45°-tilted-view SEM images of the Si nanostructures fabricated with spin-coated Ag ink having different ink ratios. The corresponding cross-sectional SEM images are also shown in the insets. ICP etching was carried out at an RF power of 75 W for 10 min in a SiCl_4_ plasma without adding Ar gas. It is clearly seen that the distribution of the fabricated Si nanostructures depends on the distribution of Ag nanoparticles (i.e., the Ag ink ratio). Also, as the Ag ink ratio was decreased, the distance between adjacent Si nanostructures decreased. From the SEM images, we estimated that the average distance between the apexes of the Si nanostructures fabricated using Ag ink ratios of 25% and 35% is less than approximately 500 nm, which is appropriate for achieving broadband antireflection according to RCWA simulations. The fabricated Si nanostructures had a tapered feature because the Ag nanoparticles were eroded during the ICP etching process from the edges of the nanoparticles. It is also seen that the top diameter of the Si nanostructures decreased as the Ag ink ratio was decreased. This was because the smaller and thinner Ag nanoparticles eroded more quickly during dry etching. As a result, the Si nanostructures fabricated using a Ag ink ratio of 25% had an average height of 236 ± 151 nm, which is much lower than that fabricated by Ag ink ratio of 35% (372 ± 36 nm) and 50% (363 ± 25 nm), and resulted in the formation of collapsed nanostructures.

The hemispherical reflectance spectra of the fabricated Si nanostructures for various Ag ink ratios in the wavelength range of 300 to 1100 nm are shown in Figure 3b. The hemispherical reflectance spectra were measured using a UV/VIS-NIR spectrophotometer (Cary 500, Varian, Inc., Palo Alto, CA, USA) with an integrating sphere kept at a near-normal incident angle of 8°. The reflection spectrum of bulk Si with an average reflectance of 36.8% is also included for comparison. It is evident that the Si nanostructures drastically reduced the reflection compared to that of the bulk Si over the entire wavelength range considered. The reflection minima shifts from the short-wavelength region to the long-wavelength region with an increasing Ag ink ratio (i.e., increasing the distance between adjacent Si nanostructures) as can be seen in Figure 1a [6,8]. The Si nanostructures fabricated using an Ag ink ratio of 25%, 35%, and 50% showed an average reflectance of 6.4%, 8.5%, and 9.6%, respectively. This result indicates that controlling the Ag ink ratio is crucial to fabricate antireflective Si nanostructures having desirable antireflection properties. Although the Si nanostructures fabricated using Ag ink ratio of 25% exhibited the lowest average reflectance among the ones fabricated with three different Ag ink ratios, a 25% ink ratio resulted in the formation of too thin nanoparticles which were unable to withstand harsh etching conditions and long etching duration, as a result producing collapsed Si nanostructures. Therefore, Ag ink ratio of 35% was chosen to form Ag nanoparticles for the reminder of experiments.

**Figure 4:**
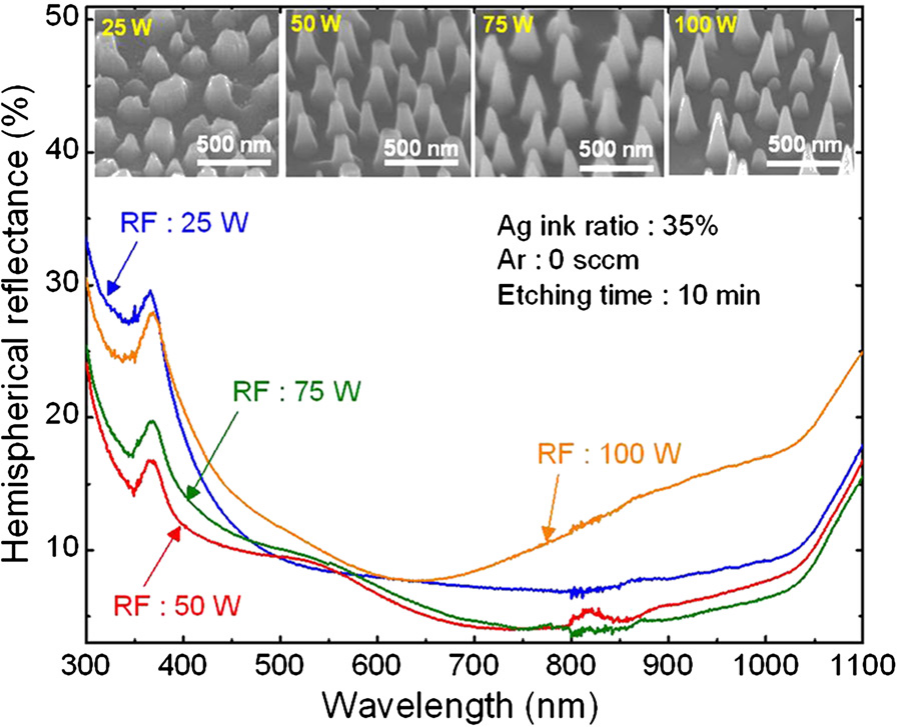
**SEM images of the Si nanostructures and the measured hemispherical reflectance spectra.** Hemispherical reflectance spectra of the Si nanostructures fabricated under different RF powers of 25, 50, 75, and 100 W using spin-coated Ag nanoparticles as the etch mask. The insets show the corresponding 45°-tilted-view SEM images.

The RF power is also an important parameter that should be adjusted to obtain Si nanostructures having the correct etching profile with broadband antireflection characteristics. Figure 4 shows the effect of RF power on the reflectance of Si nanostructures fabricated using an Ag ink ratio of 35%. The ICP etching process was carried out for 10 min with different RF powers of 25, 50, 75, and 100 W without adding Ar gas. A 45°-tilted-view SEM images of the corresponding Si nanostructures are also shown in the insets. From the SEM images, it is clear that the RF power affects the height and distribution of the Si nanostructures. As the RF power was increased, the average height of the resulting Si nanostructures first increased from 194 ± 20 to 372 ± 36 nm up to an RF power of 75 W and then decreased (286 ± 166 nm) as the RF power was further increased to 100 W. This is because at higher RF powers, the ion energy that was applied to Si surface and Ag nanoparticles was increased excessively causing the removal of thin and small Ag nanoparticles during the ICP etching process. Thus, higher RF powers resulted in the collapse of the nanostructures [8]. For this reason, at an RF power of 75 W, the formed Si nanostructures partially collapsed, and the collapse of the Si nanostructures was even more at an RF power of 100 W. Since the fabricated Si nanostructures have different heights and distances between adjacent nanostructures depending on RF power, the measured reflectance spectra of the resulting Si nanostructures are somewhat different from each other. Among four different samples, the Si nanostructures fabricated using an RF power of 50 W had an average height of 300 ± 29 nm and had the lowest average reflectance of 8.3%. Therefore, 50 W was chosen as the ideal RF power to fabricate Si nanostructures for the remainder of experiments.

**Figure 5:**
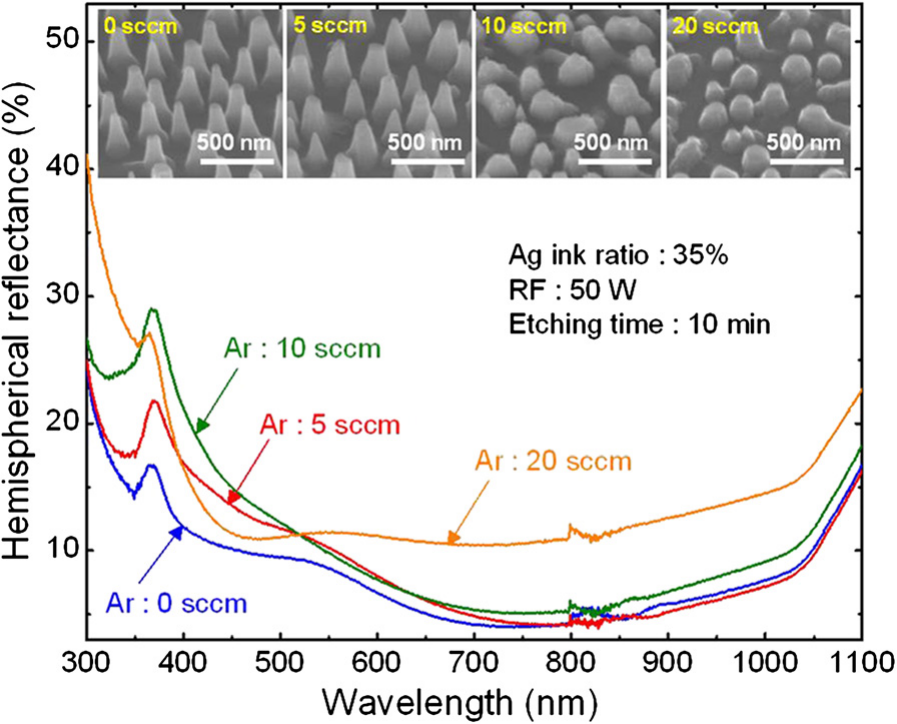
**SEM images of the Si nanostructures and measured the hemispherical reflectance spectra.** Hemispherical reflectance spectra of the Si nanostructures fabricated under different Ar flow rates of 0, 5, 10, and 20 sccm. The insets show the corresponding SEM images with a 45°-tilted view.

Another important parameter that can influence the etching profile as well as the height of the fabricated nanostructures, and therefore their reflectance, is the gas flow rate of the etchant gas mixtures. In our experiments, the flow rate for SiCl_4_ was fixed, and the influence of addition of Ar on the antireflective properties was therefore studied. Figure 5 shows the hemispherical reflectance spectra of the Si nanostructures fabricated without and with Ar gas (5, 10, and 20 sccm) for 10 min. The 45°-tilted-view SEM images of the respective Si nanostructures are also shown in the insets. As the Ar flow rate was increased from 0 to 20 sccm, the etching rate of Si nanostructures decreased from 30 to 11 nm/min, and the average height of the Si nanostructures decreased from 300 ± 29 to 110 ± 10 nm. This is attributed to the inhibition of the etching of the etching reactants by the addition of Ar to SiCl_4_ gas. With the decrease in the height, the average reflectance of the Si nanostructures increased from 8.3% to 14.4%. This experimental observation that the reflectance of the Si nanostructures increases with decrease in their height is indeed consistent with our RCWA calculations as shown in Figure 1b. This result therefore demonstrates that the addition of Ar gas is not necessary to fabricate broadband antireflective Si nanostructures.

**Figure 6:**
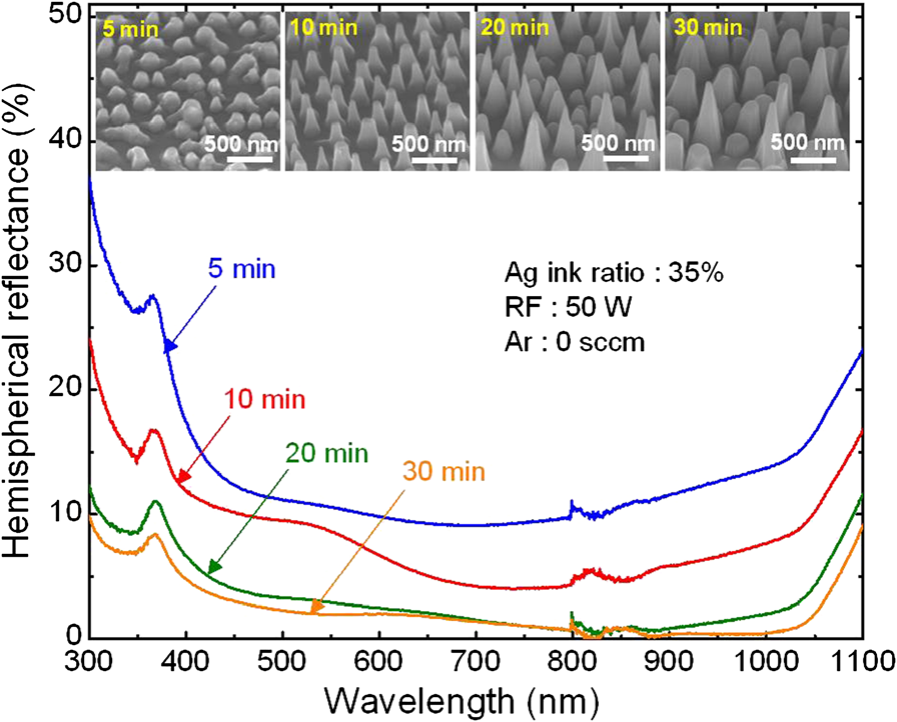
**SEM images of the Si nanostructures and measured hemispherical reflectance spectra.** Hemispherical reflectance spectra of the Si nanostructures fabricated using spin-coated Ag nanoparticles with different etching times of 5, 10, 20, and 30 min. The insets show the corresponding 45°-tilted-view SEM images.

The ICP etching time can also be adjusted to obtain the proper etching profile and optimum height to fabricate Si nanostructures having desirable antireflection properties. Figure 6 shows the hemispherical reflectance spectra of the fabricated Si nanostructures as a function of etching time, and the insets show SEM images of the 45°-tilted view of the corresponding Si nanostructures. The average reflectance of the Si nanostructures decreased from 13.7% to 2.9% when the etching time was increased from 5 to 30 min. This is because the average height of the Si nanostructures increased from 151 ± 25 to 790 ± 400 nm with increase in etching duration. This indicates that by adjusting the etching time, the height of the formed nanostructures can be adjusted, so as to tailor their reflectance behavior. However, the formed Si nanostructures partially collapsed when the etching time was 20 and 30 min. Although increasing the etching time results in nanostructures having low average reflectance, it destroys the formed nanostructures because the Ag nanoparticles which act as the etch mask are completely removed with increasing etching time. In addition, a too tall height of the nanostructures made them mechanically unstable, making them impractical to be used. Therefore, an Ag ink ratio of 35% and ICP etching conditions such as 50-W RF power, 0-W ICP power, and 2-mTorr process pressure for 10 min without adding Ar gas in a SiCl_4_ plasma are the optimum process conditions suitable to produce antireflective Si nanostructures having broadband antireflective features using the proposed technique.

**Figure 7:**
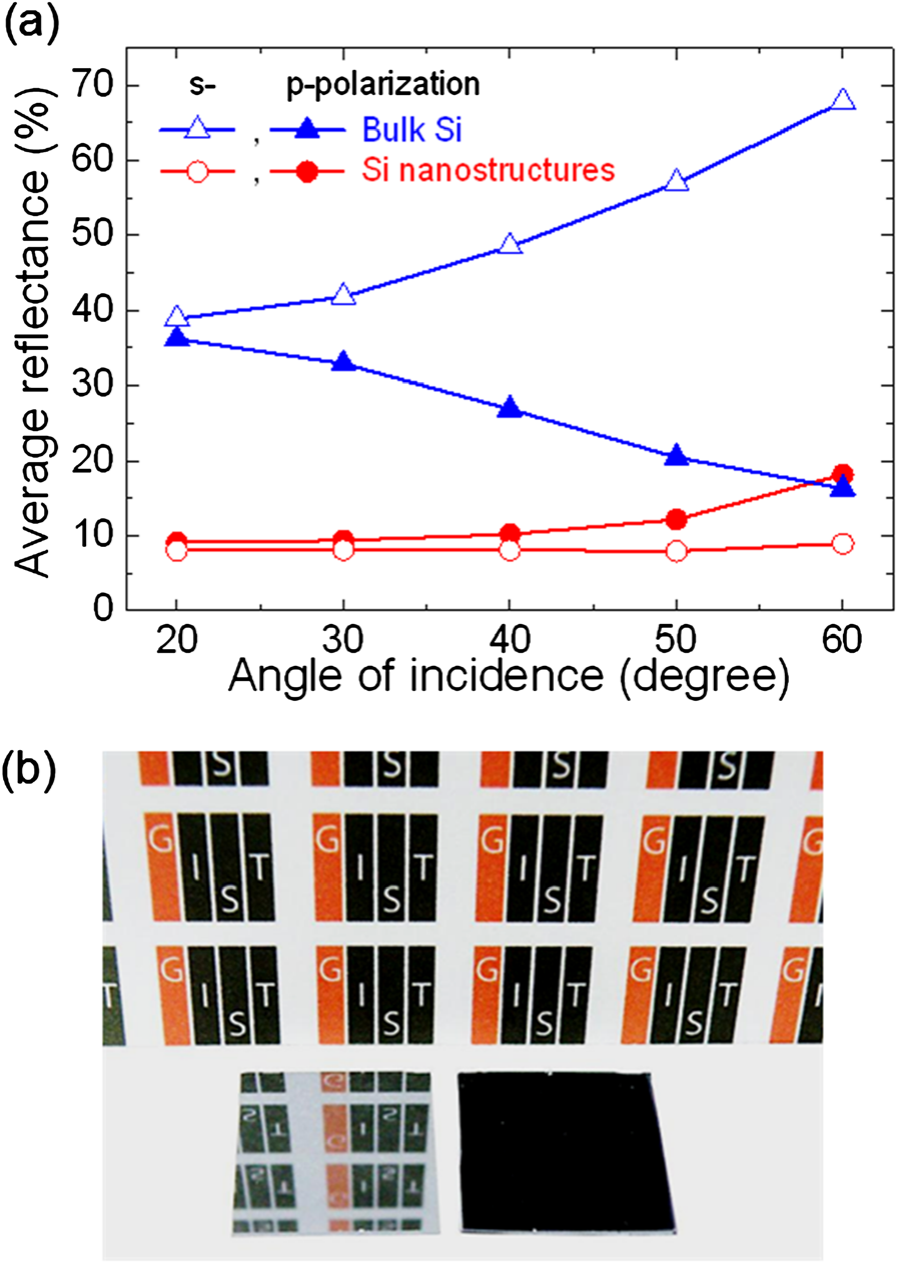
**Incident angle-dependent average reflectance and photographs of bulk Si and Si nanostructures. (a)** Average reflectance as a function of incident angle for s- and p-polarized light and **(b)** photographs of bulk Si (left) and Si nanostructures (right) fabricated using the optimum fabrication conditions.

Incident angle- and polarization-dependent antireflection properties are also important parameters used to evaluate the effectiveness of antireflectors [13]. For a good antireflector, the reflection over a wide range of light incident angles should be as low as possible for both s- and p-polarized light. Figure 7a shows the incident angle-dependent average reflectance of the Si nanostructures fabricated using the optimum process conditions and the bulk Si for polarized light. The incident angle-dependent reflectance was obtained using a Cary variable angle specular reflectance accessory in specular mode. It is clearly seen that the bulk Si has a high reflectance, and the reflectance is highly sensitive for both s- and p-polarized incident light for a wide range of incident angles. In contrast, the fabricated Si nanostructures show almost polarization-independent antireflection property over a wide range of incident angles. The photographs of bulk Si and antireflective Si fabricated by the optimum process conditions are displayed in Figure 7b. As can be seen, bulk Si has poor antireflective properties, and hence, the reflected background image can be seen. On the other hand, Si nanostructures do not reflect the background image and display a black surface, demonstrating its superior antireflection property.

## Conclusions

We fabricated antireflective Si nanostructures by a simple nanofabrication technique using spin-coated Ag nanoparticles and a subsequent ICP etching process. Theoretical investigations based on RCWA method were carried out prior to fabrication to determine the effect of variations in height and period on the antireflection properties of Si nanostructures. Using the results from RCWA as a guideline, various Si nanostructures with different distribution, period, and height were fabricated by adjusting the Ag ink ratio and ICP etching conditions. It was found that the fabricated Si nanostructures significantly reduced the surface reflection losses compared to bulk Si over a broad wavelength range. Si nanostructures fabricated using a 35% Ag ink ratio and optimum ICP etching conditions showed excellent antireflection properties over a broad wavelength range as well as polarization- and angle-independent reflection properties. The antireflective Si nanostructures fabricated using this simple, fast, and cost-effective nanofabrication technique exhibits great potential for practical Si-based device applications where light reflection has to be minimized.

## Competing interests

The authors declare that they do not have competing interests.

## Authors’ contributions

JBK carried out most of the experimental works associated with fabrication and characterization of samples, analyzed the results, and prepared the manuscript. CIY proposed the original idea and helped in preparing the manuscript. YHL helped in fabrication and characterization of samples. SR helped in characterization of samples and preparation of the manuscript. YTL developed the conceptual framework and supervised the whole work, and finalized the manuscript. All the authors read and approved the final manuscript.
